# Soft, Modular Power for Composing Robots with Embodied Energy

**DOI:** 10.1002/adma.202414872

**Published:** 2025-01-02

**Authors:** Chong‐Chan Kim, Anunth Rao Ramaswami, Robert F. Shepherd

**Affiliations:** ^1^ Department of Mechanical and Aerospace Engineering Cornell University 124 Hoy Road Ithaca NY 14850 USA

**Keywords:** 3D printing, dry adhesion, embodied energy, modular structure, redox flow battery, untethered soft robots

## Abstract

The adaptable, modular structure of muscles, combined with their confluent energy storage allows for numerous architectures found in nature: trunks, tongues, and tentacles to name some more complex ones. To provide an artificial analog to this biological soft muscle, a self‐powered, soft hydrostat actuator is presented. As an example of how to use these modules, a worm robot is assembled where the near totality of the body stores electrochemical potential. The robot exhibits an extremely high system energy density (51.3 J g^−1^), using a redox flow battery motif, with a long theoretical operational range of more than 100 m on a single charge. The innovation lies in the battery pouch, fabricated with a dry‐adhesion method, automatically bonding Nafion separators to a silicone‐urethane copolymer body. These pouches contain anolyte within a hydrostat pod filled with catholyte, increasing current density per pod. Each pod has a motor and tendon actuator for radial compression and expansion. By linking these self‐contained pods in series, the robot worm is created that automatically navigates an enclosed, curved path. This high‐capacity soft worm also climbs up and down a vertical pipe, using a two‐anchor crawling gait, with an extra payload equivalent to 1.5 times its body weight.

## Graphical abstract

A worm‐like robot composed of modular, self‐contained actuators is developed. The modules with embodied zinc‐iodide redox flow batteries are fabricated using a novel bonding method, allowing for reliable power supply over extended periods more than 35 h. Each module operates independently to create various gait patterns, demonstrating efficient locomotion.

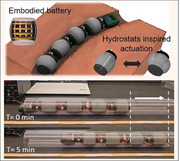

## 1 Introduction

As the demand for increased agility in robotics grows, their complexity will likely also increase—with increasing densities of degrees of freedom (DoF) and sensing. With these increased DoF's will probably also come increased power consumption; energy capacity will become more important.^[^ ^1^ ^]^

A module that can be used to rapidly compose and iterate robots with increased agility, while commensurately adding energy capacity could be an important component in future robotic systems. Modularity in engineering refers to a design approach where a system is divided into multiple independent parts. Constructing complex machines is made easier using modular design approaches, as fewer decisions need to be made regarding specific and, sometimes, more optimal configurations; and, most importantly, repair of damaged components is more simple. Using modular design, new, complex machines can be built faster, because there is less time between each iteration.^[^ ^2^, ^3^ ^]^

Muscles provide a good biological example of a self‐contained actuator module. In a relatively simple arrangement, antagonistic pairs of skeletal muscle modules (e.g., bicep and tricep) extend and retract our arms. Tongues, trunks, and tentacles^[^ ^4^ ^]^ are more complex; muscular hydrostats composed of longitudinal, transverse, and oblique striated muscle groups. These muscle groups exert forces in different directions, enabling various movements such as bending, extension, and torsion.^[^ ^5^ ^]^ The glycogen stored in muscles enables muscles to function without relying immediately on external sources. Every kilogram of human muscle stores about 15 g of glycogen, which is equivalent to approximately 1200‐1600 kcal of energy for the whole human body (≈88.2 J g _body weight_^−1^).^[^ ^6^, ^7^ ^]^ Where there be muscle, there be energy.

Soft robots, in particular, stand to benefit from self‐powered actuation modules akin to natural muscle. Their use, particularly beneficial for situations that benefit from continuum body deformations (e.g., swimming,^[^ ^8^, ^9^, ^10^, ^11^ ^]^ crawling,^[^ ^12^, ^13^, ^14^, ^15^, ^16^, ^17^, ^18^, ^19^, ^20^, ^21^, ^22^ ^]^ dexterous manipulation),^[^ ^23^, ^24^, ^25^, ^26^, ^27^, ^28^ ^]^ in the majority of cases relies on power tethers (e.g., pneumatic,^[^ ^9^, ^15^, ^16^, ^17^, ^18^ ^]^ hydraulic,^[^ ^27^, ^28^ ^]^ electric,^[^ ^10^, ^14^, ^19^, ^26^ ^]^ combustive)^[^ ^29^, ^30^ ^]^ for locomotion; particularly when not used in underwater applications that support them buoyantly. These tethers simplify the structure of the robot by placing bulky and rigid components such as power sources, pumps, and motors externally, to maintain the softness in the robot. The tethered approach, however, significantly limits the operational range and navigational ability of the robot. Although untethered soft robots operating using onboard components have been developed, the untethered robots still suffer from short operational lifetime duration due to the limited energy capacity that they can carry on themselves.^[^ ^8^, ^12^, ^13^, ^14^, ^20^, ^21^, ^22^ ^]^ Moreover, untethered robots often require sacrificing a significant portion of the system to rigid components.

Flexible power sources that can endure mechanical deformation have been extensively studied for use in soft robotics and wearable devices. These include Li‐ion batteries,^[^ ^31^, ^32^, ^33^ ^]^ Zinc based batteries,^[^ ^34^, ^35^, ^36^, ^37^, ^38^ ^]^ solar cells,^[^ ^39^ ^]^ supercapacitors,^[^ ^40^ ^]^ microbial fuel cells,^[^ ^41^ ^]^ and energy harvester.^[^ ^42^ ^]^ Such flexible power devices can be realized using intrinsically flexible materials or through structural approaches, such as rigid islands or buckles with pre‐stretching. The flexibility of these power sources, however, often results in significant performance degradation and limited energy storage capacity (Table S1, Supporting Information). Additionally, although flexible power sources offer the advantage of being easily integrated into systems, they still require additional space to accommodate them.

Embodied energy, a design strategy that improves the system level of energy density through the multifunctional use of embedded power sources, has recently been introduced.^[^ ^37^, ^43^ ^]^ Redox flow batteries (RFB) that were integrated into the soft robotic system have demonstrated the ability to provide sufficient power and capacity, while showing their intrinsic compliance, attributed to the storage of electrical energy in liquid form. One of the challenges for the fabrication of the RFB cell, however, is a hermetic sealing around the perimeter of the ion exchange membrane that is required to prevent leakage or cross‐mixing of the electrolytes. Aubin et al.^[^ ^37^ ^]^ revealed that a number of small leaks emerged at the junction where the Nafion and polymer were bonded, necessitating further remediation steps. A more reliable sealing method would streamline the manufacturing process and enable the production of batteries in diverse form factors. This consideration is particularly crucial for soft robots, which inevitably undergo continuous deformation

Here, we present an untethered terrestrial crawling robot that uses this technique to have an integrated modular power system throughout its soft body. Each module serves as an actuation unit driving locomotion gaits and functions as an independent deformable battery cell providing a standard voltage of 1.3 V and an energy capacity of 3387 mWh. Each pod is composed of a stack of anolyte pouches immersed in catholyte, yielding high areal capacity (119 mAh cm^−2^, *A*_POD_ = 32 cm^2^), and large energy density of 51.3 J g^−1^ for the entire system, that theoretically operates for ≈35.6 h of operation time and 105 m of travel distance for a single charging. We demonstrated the modularity and capacity of these pods by assembling a linear array, a worm, of them and drove them, untethered, vertically against gravity and in circles within enclosed spaces.

## 2 The Self‐Powered Terrestrial Soft Robot

**Figure 1:**
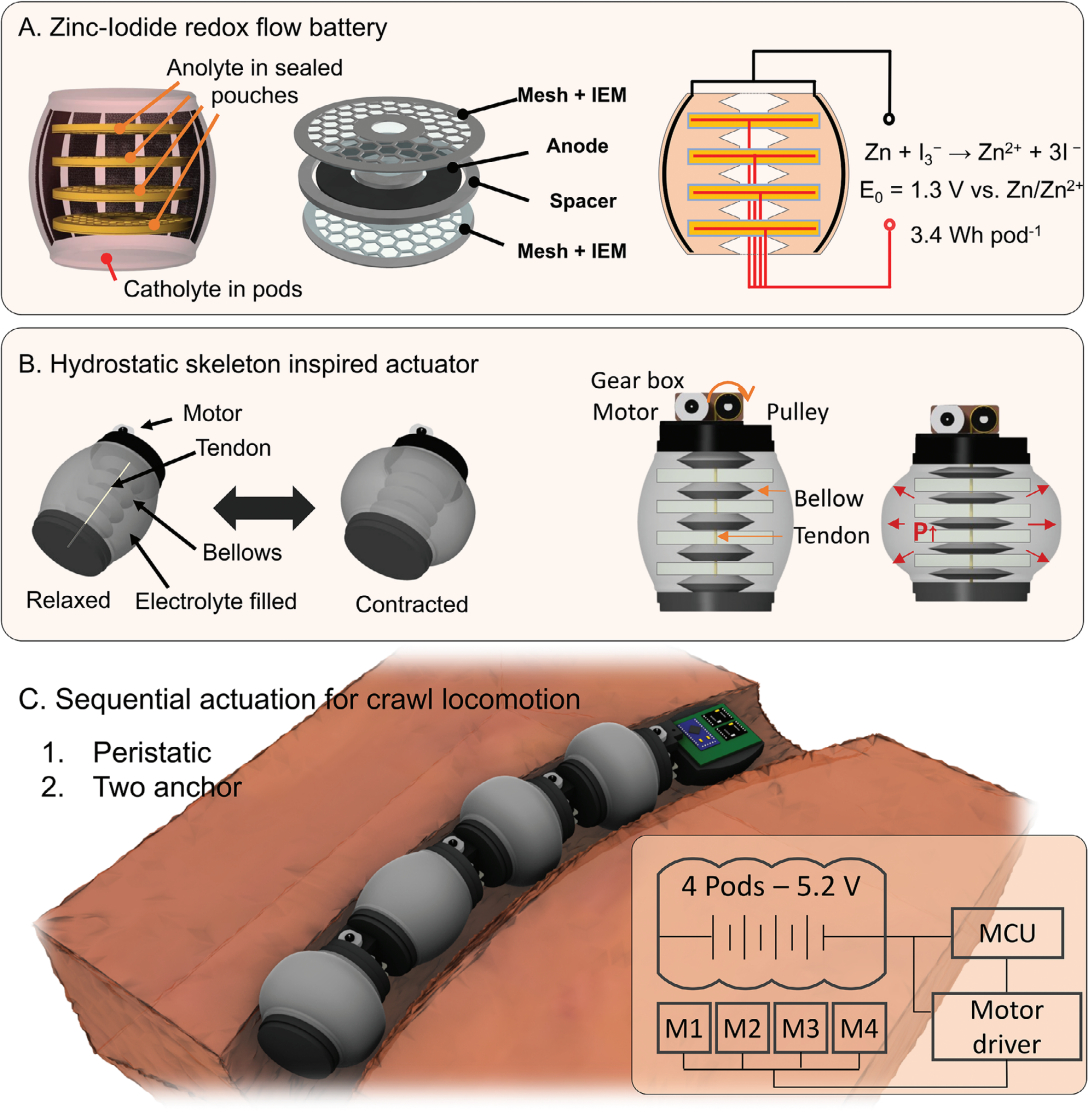
Modular pod for self‐contained actuation in soft robots. A) The pod is an independent modular unit that includes an actuation mechanism and energy entities. The zinc‐iodide redox flow battery, standard voltage of 1.3 V, integrated into each pod self‐powers the entire system. The amount of catholyte filling the pod and the anode area in the inner pouches enable the high capacity of the battery (118 mAh cm^−2^, 3387 mWh). B) The actuation mechanism of the pod mimics a hydrostatic skeleton of a worm, creating radial expansion by axial contraction of an incompressible body. A DC motor and a tendon in each pod drive the full contraction (*ΔL/L* = ≈10%) within 1.8 s, achieving rapid actuation. C) The modularity of the pods enables easy reconfiguration and independent control of contraction and expansion. A series of four connected pods generated various patterns of sequential actuation for effective forward motion, adaptable as needed. Peristaltic locomotion and two‐anchor locomotion were demonstrated on a flat surface and inside a pipe, respectively.

The untethered terrestrial soft robot, depicted in **Figure** **1**, is designed to operate within a constrained environment, such as narrow tunnels underground or within pipelines, which imposes limitations on the shape and gait of the robot. The robot has a slender (diameter, *D* = 60 mm) and long (length, *L* = 350 mm) body, consisting of four actuation modules, pod, integrated with a zinc‐iodide redox flow battery and one control module at the end of the actuation modules. The power for actuation and control was entirely provided by the zinc‐iodide redox flow batteries embodied within each pod (Figure 1A). The interior of the pod is filled with catholyte, within which there are pouches made of ion‐exchange membranes (IEM). These pouches create separate compartments, preventing mixing with the catholyte and providing a designated area for the anode. The pod achieves a high electrical capacity of 3.4 Wh per pod, facilitated by the catholyte filling the pod and the multiple pouches providing the anode area necessary for zinc electrodeposition during charging. The locomotion principle for the actuation module is inspired by hydrostatic skeletons, as observed in earthworms. The actuation mechanism is depicted in Figure 1B. We used a tendon‐driven mechanism that allowed compact, independent control of each body segment with rapid actuation. Longitudinal contraction increases the pressure of the body filled with an incompressible fluid, inducing its radial expansion. The resilience of the body, absorbing elastic energy in the stretchable exterior body shell during longitudinal contractions, returns it to its original shape, allowing for passive longitudinal extensions. The four pods are connected in series to power the controller and motor drivers, while the actuation of each pod is independently controlled (Figure 1C). This setup enables a variety of gait patterns, allowing the robot to demonstrate efficient locomotion tailored to different environments.

## 3 Pouch Anode Cell

**Figure 2:**
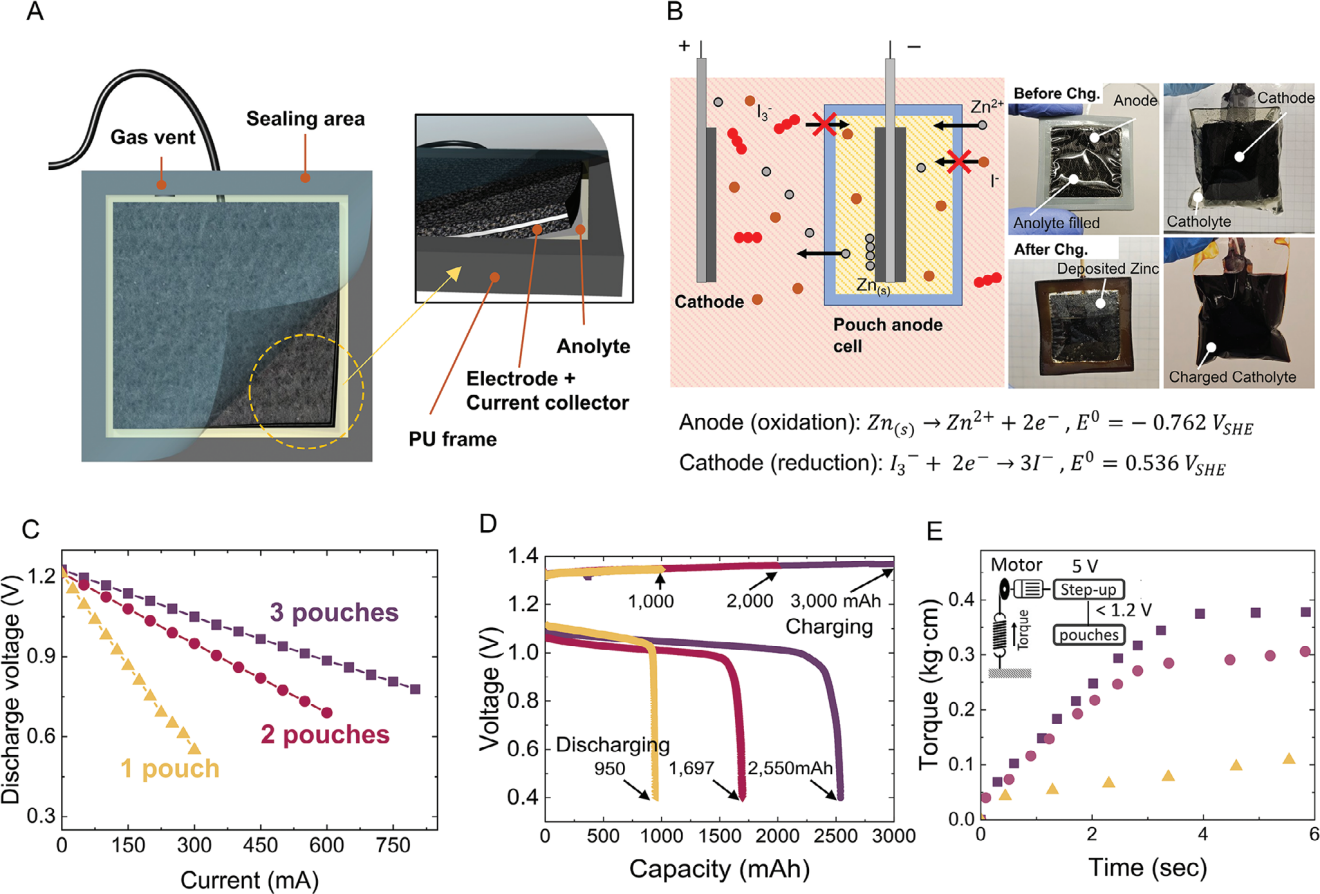
Demonstration and characterization of the pouch anode cell. A) Schematic of the pouch anode cell containing the anode composite within a sealed enclosure made of ion‐exchange membranes. The anode composite consists of a stainless steel mesh (200 wires per inch) sandwiched between two layers of 0.35 mm carbon cloth. An 8 cm^2^ anode was used, matching the size of the anode in the worm robot. The gas vent at the top of the frame improved the cyclic performance by releasing accumulated hydrogen. B) The ion‐exchange membrane, which constitutes the top and bottom faces of the anode pouch, prevents the mixing of catholyte and anolyte while allowing the passage of Zn^2+^ during charge and discharge. During charging, zinc ions from the catholyte migrate into the pouch and deposit as metallic zinc on the anode surface, while oxidized iodide ions form triiodide, giving the solution a deep red color (bottom left and right in inset). C) Polarization curve for the multiple pouches. Multiple pouches share the catholyte and function as a battery connected in parallel. D) Capacity curve for the multiple pouches. Multiple pouches provide the surface area, which is the bottleneck for capacity, thereby enabling greater overall capacity. E) Torque on the motor powered by the multiple pouches. Multiple pouches supplied greater power to the motor, enabling it to generate higher torque.

We devised a pouch‐like anode cell for a zinc‐iodide redox flow battery that can accommodate more electrodes within a system to increase power capability and energy capacity. Since the active species in the redox flow cell is mobile, the anode and cathode need to be separated in a hermetically sealed chamber. The pouch anode enclosure, shown in **Figure** **2**A, includes the anode and the hermetic enclosure separating the anode from the cathode. We designed the pouches used in the robot as disk shapes with a central hole. For a simplified demonstration of pouch operation, however, we fabricated straightforward square‐shaped pouches. We set the internal anode size to 8 cm^2^, matching the anode used in the robot. This electrode area was determined by considering the factors necessary for robot operation and control, as described in the power requirement estimation.

The pouch consists of two layers of ion exchange membranes (Nafion211, DuPont), the silicone polyurethane frame, and a flexible anode composite. We placed the flexible anode composite made out of stainless‐steel mesh and carbon cloth in the center of the frame and threaded its terminal wire through the frame. We affixed the ion exchange membranes to both the top and bottom surfaces of the frame, using a new adhesion technique, the details of which will be discussed later in this study, thereby creating a sealed compartment. The interior space of the pouch was filled with an anolyte. We placed a one‐way release valve on the frame, designed to ventilate hydrogen gases that accumulate during battery operation (Figure S1, Supporting Information).^[^ ^44^ ^]^ The pressure accumulated inside the pouch pushed the anolyte outward, resulting in internal dehydration and a reduction in cyclic performance. Pressure relief improved cyclic performance ten times compared to our prior work.^[^ ^37^ ^]^

The pouch is sealed, allowing us to immerse it in the catholyte chamber, while the anode and cathode are coupled through the ion exchange membrane, creating a full cell for the electrochemical reactions (Figure 2B). We placed the pouch anode cells and cathodes into plastic bags containing catholyte and performed charge‐discharge cycles. The bonding between the ion exchange membrane and polyurethane that we used provided secure sealing, and even after cycling, the clear anolyte confirmed that there was no cross‐mixing with the catholyte (Figure S2, Supporting Information).

The pouch configuration enhances the design flexibility of the soft robot, allowing it to undergo significant deformation during actuation, by placing it within the inner volume of the pod. The pouch configuration also enables easy stacking in a single cathode chamber; as the pouch comes equipped with its own sealed chamber, it can be stacked within a single cathode chamber without the need for additional components.

Adding more pouches increases the active area of the electrodes participating in the electrochemical reaction, resulting in higher current output. Furthermore, with more pouches we can expect greater battery capacity as the electrodes contained within them provide more surface area for the electrodeposition of zinc during charging cycles. To understand the power that a multi‐pouch pod can deliver, we measured how the cell voltage changes with current output as shown in Figure 2C. The maximum power is attained at half of the initial cell voltage, 0.65 V for a Zinc‐iodide cell as per the maximum power transfer theorem. However, we targeted 0.9 V for discharging voltage during the operation of the robot considering that withdrawing the maximum power dissipates half of the energy at the internal resistance of the battery. Moreover, the discharging voltage of 0.65 V inevitably requires a voltage boost converter, introducing additional power losses. At the target voltage of 0.9 V, a single pouch provided a current output of 135 mA, and it increases proportionally with the number of pouches.

We characterized the scalability of the capacity using multiple pouches (Figure 2D). A single pouch demonstrated a high areal capacity (119 mAh cm^−2^), resulting in 953 mAh of discharge capacity for 1000 mAh of charge with a good cyclic performance (Figure S1, Supporting Information). We attribute this high capacity from the accessibility of both the front and back sides of the anode in the pouches for the electrochemical reaction. The addition of the pouches scaled up the capacity of the system by providing an active area for zinc deposition. The Coulombic efficiency, the ratio of the discharge and charge capacity, however, decreased with the addition of the pouches, but the total capacity for two and three pouches increased to 1697 and 2540 mAh, respectively.

The measured torque on the motor indicated the actuation capability of the multiple pouches (Figure 2E). Even though multiple parallel‐connected pouches can provide higher power, the torque generated by the motor is limited by the input voltage. As the voltage of multiple pouches remains the same as that of a single pouch, we stepped up the voltage of the parallel connected pouch cells to 5V using a voltage boost converter to power the motors. A single pouch could not keep the minimum input voltage required of the voltage boost converter. In the case of more than two pouches, however, they delivered greater power to the motor, resulting in a torque of 0.38 kg∙cm with three pouches.

## 4 Dry‐Adhesion on Ion Exchange Membranes

**Figure 3:**
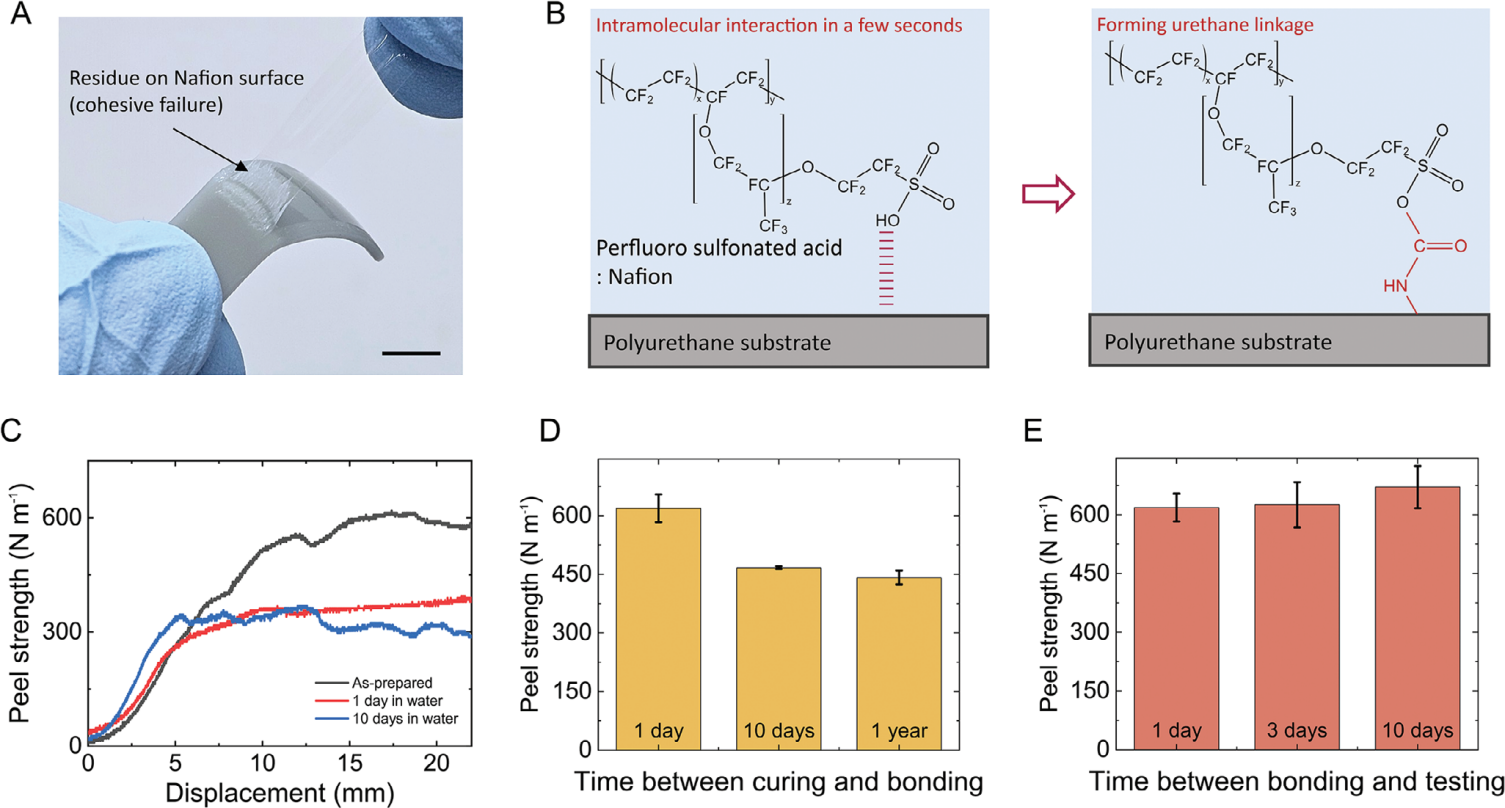
Mechanism and performance of the dry‐adhesion between Nafion and polyurethane. A) Photograph of the adhesion surface showing cohesive failure on polyurethane substrate, The scale bar is 10 mm. B) Upon contact, the adhesion forms instantly by intramolecular interaction such as hydrogen bond or electrostatic interaction, followed by the urethan linkage attributed to a stable bond. C) Force–displacement curve measured using T‐peel test for the adhesion samples; as‐prepared (black), immersed in D.I. water for 1 d (red), and 10 d (blue). The specimen width was 1 cm, and the crosshead speed of the tensile test was set to 0.05 mm s^−1^ to minimize viscoelastic effects and localized stress concentrations. D) The duration of surface reactivity in cured polyurethane substrates was tested. Polyurethane substrates were fully cured using a normal curing process, which involved UV 3D printing followed by thermal curing at 120 °C. After curing, the substrates were stored at room temperature for different durations—1 d, 10 d, and 1 year—before bonding tests were conducted. E) Stability of the adhesion over time (1 d, 3 d, and 10 d) after the bonding. Once the bond was formed, no decrease in bonding strength was observed over time. (Error bars indicate SDs, *n* = 3).

We discovered a dry‐adhesion method to adhere Nafion and polyurethane rubber, and used it to fabricate the pouches. The as‐received Nafion membrane and fully cured polyurethane surface formed a strong bond within a few seconds without additional surface treatment or adhesive, facilitating rapid fabrication processes for the pouches (**Figure** **3**A; Video S1, Supporting Information). Since the adhesion strength was stronger compared to the yield stress of the materials, failure occurred in the elastomer—with residue remaining on the Nafion surface as shown in Figure 3A. We confirmed, through Raman spectroscopy analysis of the bonding surface, that the residue on Nafion membrane is polyurethane and that no new bonding, other than the urethane linkage, has formed (Figure S3, Supporting Information). We have tested the adhesion with a commercial polyurethane rubber based on toluene diisocyanate (TDI) prepolymer (Vytaflex and Reoflex, Smooth‐on), and they also showed a strong dry‐bond with the Nafion (Figure S4, Supporting Information). The Nafion is known for its chemical inertness because of its polytetrafluoroethylene (PTFE) backbone, but a plausible mechanism for this adhesion is that the sulfonic acid groups on the Nafion form intramolecular interactions (hydrogen bond or electrostatic interaction) with the polyurethane surface, contributing to forming a bond in milliseconds. In the following step, the hydroxyl group on Nafion may react with the isocyanates on the polyurethane surface, forming a urethane linkage.^[^ ^45^ ^]^ Further analysis, however, is needed to validate the bonding mechanism. The adhesion strength of the bond between the Nafion membrane and the 3D‐printed silicone polyurethane is high, as measured using a T‐peel test (Figure 3C). Specifically, we measured a peel strength of *Γ* ≈ 620 N m^−1^ at the plateau region, which is a comparable peel strength with 3M duct tapes (*Γ* ≈ 600N m^−1^). We demonstrated the stability of the adhesion in aqueous environments, as the Nafion is immersed in electrolyte solutions during use. When immersed in deionized water for one day, the peel strength was reduced to *Γ* ≈ 362 N m^−1^. This reduction we attribute to polar water molecules screening hydrogen bonds. We did not, however, observe further reduction over a 10 d immersion, likely attributed to our proposed stable covalent bond on the adhesion surface. We also observed that the bonding exhibits humidity dependency (Figure S5, Supporting Information). The sulfonic acid groups in Nafion interact with water molecules to form hydronium ions, which absorb surrounding water molecules to create clusters and strengthen hydrogen bonds.^[^ ^46^ ^]^ At low humidity, these water clusters shrink, weakening the hydrogen bond network within Nafion, which can lead to reduced adhesion. This serves as evidence that hydrogen bonds play a crucial role in mediating the formation of the bond.

Furthermore, in dry testing, we determined that the reactivity of the polyurethane surface for good adhesion lasted a long period after the curing of polyurethane, making it easier to manufacture pouches for the pods (Figure 3D). The polyurethane sample we cured 10 d prior to adhesion showed a slight drop to *Γ* ≈ 450 Nm^−1^ compared to immediate bonding, but a specimen bonded 1 year post curing showed a peel strength of *Γ* ≈ 442 N m^−1^ post bonding. We also evaluated the stability of the adhesion for increasing bonding times. Figure 3E shows that once the bonding was created, the peel strength of the surfaces did not significantly change over time.

## 5 Pod Characterization

**Figure 4:**
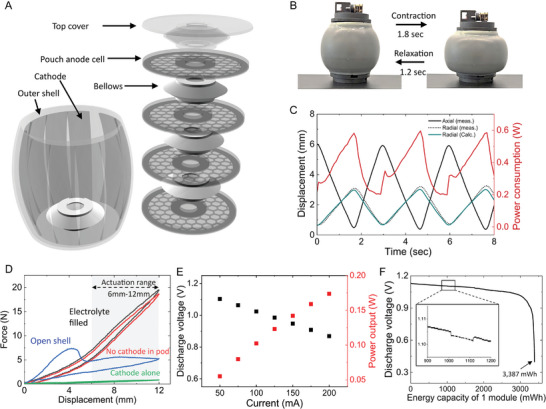
Mechanical and electrochemical characterization of the pod. A) The exploded view of the pod. The pod includes four anode pouches in the center that is surrounded around by the cathode. Inside and outside of the pod was filled with 3 m zinc iodide solution. B) The hydrostatic skeleton inspired actuation module, axial contraction of the pod, driven by a tendon‐motor mechanism, results in radial expansion due to the presence of an incompressible electrolyte. C) Dimension changes and power consumption during actuation. The peak power was 0.59 W at full contraction, while in the passive extension phase, which used stored elastic energy, power consumption dropped to about 0.2 W, the minimum required for control. D) Compressive loading and unloading curves of the pod. The force required for a 6 mm stroke, from 6 mm to 12 mm including preloading, was 19.5 N. The internal battery components, arranged to minimize interference with pod motion, required only an additional 1 N for contraction. E) Polarization curve of the single module integrated with four pouches. Upon completing all integrations, a single pod produced approximately 0.175 W at the target voltage of 0.9 V, and the total power of the robot that consists of four pods was confirmed to exceed 0.7 W. F) Total discharged energy capacity for single actuation module. The single pod with 32 cm^2^ of anode and 60 mL of 3 m ZnI_2_ catholyte discharged 3387 mWh with 85% of Coulombic efficiency. The inset figure shows a voltage drop when the module was discharged with static mode.

We fabricated the pod with four disk‐shaped anode pouches using the new dry adhesion method we developed. The pouch consists of five layers (mesh/IEM/spacer for electrode/IEM/mesh), which are joined together through the adhesion between the ion exchange membrane and polyurethane (Figure S6, Supporting Information). The arrangement of the pouches and the shared cathode in the pod is illustrated in **Figure** **4**A. We designed the pouch cells for the actuation module as circular shapes (*D*: 42 mm) with an 8 mm diameter of hole in the center (Figure S7, Supporting Information). We placed four pouches in the middle of the pod and stacked them alternatively with the bellow‐like structure to allow longitudinal contraction. Each pouch had an electrode area of 8 cm^2^, which was determined by the Power budget optimization in the Methode section. The four anodes in the pouches and the one shared cathode (*A*_cathode_ = 54 cm^2^) surrounding the four pouches were loosely coupled through the electrolyte, allowing deformation during locomotion (Video S3, Supporting Information).

As DC motor driven tendons are a simple and efficient method to drive locomotion, we used them as the prime mover of our pods. The motor at the top of each pod caused longitudinal contraction by rotating a pulley to spool the tendon. The contraction of the pod filled with incompressible fluid increased the internal pressure, thereby causing stretching of the side wall and radial expansion. When the motor reverses direction, the pulley releases tension and the resilience of the pod's elastomeric walls causes it to elongate longitudinally. Figure 4B and Video S2 (Supporting Information) demonstrate the real time actuation of the pod with the electric power that the fully assembled robot can provide. The pod reached a fully contracted state within 1.8 s and returned to its original state within 1.2 s. In an incompressible closed cylinder that maintains a constant volume, the relation between radius and length follows the equation below.

(1) $$\frac{dr}{dl}=-\frac{r}{2l}$$

where *r* and *l* are the radius and length of the cylinder respectively. This equation indicates that a small fractional change in the shortening of the radial direction results in twice the lengthening in the axial direction. The incompressibility of the pod filled with electrolytes makes axial and radial locomotion antagonistic to each other, enabling the pod to repeat the cyclic motion. We recorded the trajectory of the height and radius changes during the actuation and the power consumption of the system, as shown in Figure 3C. As the pod axially contracted (black solid line decreased), the radius of the pod (black dot line) increased, following the calculated radius (green solid line) by Equation (1).

We measured the energy consumption during the contraction and relaxation using the area under the power curve and found it to be 0.40 J and 0.10 J, respectively, after subtracting the constant power consumption by the electronics (microcontroller unit and motor drive). The maximum and average power consumption during actuation were 0.59 and 0.38 W, respectively. We investigated the force required to compress the module and the force that the module can exert to push other modules during passive axial extension using the compressive loading–unloading test of the actuation module (Figure 4D). During the locomotion of the four pouch pod, the module was pre‐compressed 6 mm to keep the tension on the thread and actuated from 6 to 12 mm of initial height (6 mm of stroke). The force required for the fully contracted state (12 mm) was 19.5 N, implying that 1.0 kgf cm of torque on the motor is needed for the actuation, considering the radius of the pulley was 5 mm. The force on the unloading curve indicates that the module can push other modules when the module passively elongates; the resilience of the module assists actuation, resulting in a high force on the unloading curve.

We determined the direct current internal resistance (DCIR) of the battery integrated into the pod as 1.2 Ohms from the polarization curve shown in Figure 4F. The voltage drop in the polarization curve is mainly due to the Ohmic loss, attributed to the ion resistance on the path between the loosely coupled anodes and cathode. As the four pods are connected in series, the total power output of the robot (at > 3.5 V) was 0.7 W which is higher than the maximum power consumption of the motor during the actuation. Figure 4G shows the energy capacity of the single pod with four pouches. During discharging, the pod was shaken with an orbital shaker to replenish the electrolyte to the electrode surface. We measured the single pod discharge to be 3387 mWh of energy at 160 mA of discharge current (5 mA cm^−2^) and kept the discharge voltage higher than 0.9 V for 97% of discharge energy. We verified the voltage drop when the battery was discharged statically (i.e., not actuating), as the battery operates in this mode during the intervals when other modules are actuating in the locomotion gaits we used. As shown in the inset curve in Figure 4F, the voltage drop was negligible within the range of current density in which our system operates.

## 6 Operating Untethered System

**Figure 5:**
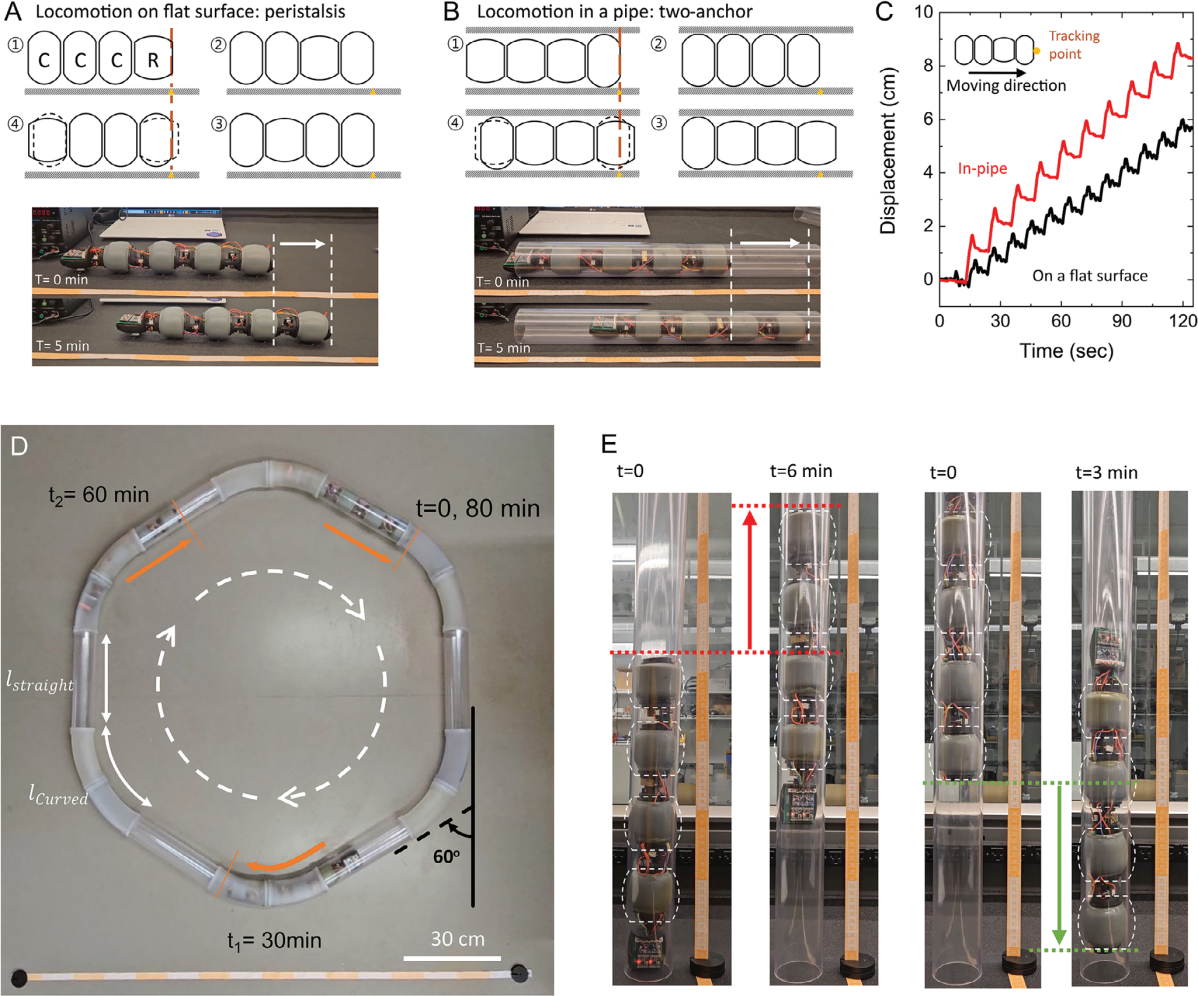
Locomotion performance of the untethered crawling robot. A,B) Demonstration of the robot on a flat surface with a peristaltic locomotion pattern and in the pipeline with a two‐anchor locomotion pattern. *R* and *C* in the schematic figure denote the relaxed and contracted state, respectively. The inner diameter of the pipe was 65 mm. C) Trajectory of the worm head during locomotion. Black curve: The stride length and cycle duration for the peristaltic locomotion pattern were 4.7 mm and 9.3 s, achieving a net speed of 30 mm min^−1^. Red curve: The stride length and cycle duration for the two‐anchor locomotion pattern were 8.2 mm and 11.3 s, achieving a net speed of 44 mm min^−1^. D) Navigating within the pipeline that has the hexagon‐shaped path combined with straight segments (*L*: 300 mm) and curved segments (*L*: 300 mm, *R*: 300 mm). The overall duration for a single round trip and distance is 1 h 20 min and 3.8 m. The energy used for the trip is estimated to be approximately 500 mWh, which accounts for about 4% of the robot's total energy capacity. E) The robot climbs up and down in the vertical pipe with 25 and 50 mm min^−1^ speed.

Using these pods, we built a robot with four of them in series and one control module as shown in Figure S8 (Supporting Information). The serial configuration allowed for the battery to provide sufficient voltage to the microcontroller and motor drive without the need for a voltage boost converter. The motor drive in the control module powered the motors on each module through the wire in the center cavity of the pods. We demonstrated the locomotion of the robot in two environments, 1) on a flat surface and 2) in a pipeline (**Figure** **5**A,B and Videos S4 and S5, Supporting Information). For locomotion on a flat surface, we used a peristaltic locomotion gait, a common mode of movement observed in many worms, where forward motion is achieved by propagating a wave of elongated bodies. Anchoring plays a crucial role in a peristaltic crawler as it prevents sliding backward while it moves the body forward. A contracted body that has an enlarged diameter contacts a bottom plane, resulting in local sliding resistance. We designed the peristaltic locomotion gait in which all pods are contracted except one relaxed pod (axially elongated) in all sequences, so we could have maximum friction from the contracted modules. Setae‐like supports were attached underneath the robot to engage anisotropic friction, aiding its locomotion (Figure S10, Supporting Information). The stride length of a single cycle of the peristaltic locomotion was *l*, where *l* denote the displacement single module makes. Figure 5C shows the trajectory of the head part of the robot. The net stride length was 4.7 mm for each single cycle and the average speed was 30 mm min^−1^.

We also demonstrated locomotion inside a pipe, a common environment in need of inspection tools. For this environment, we adopted a two‐anchor crawling gait, as the pipe walls provide frictional contact against its movement. Since radial expansion allows for strong frictional contact with the pipe wall, it serves as a useful anchor for effective movement. The stride length of a single cycle for the two‐anchor crawling is given by *(n‐*2*) × l*, where *n* is the number of the actuation modules. The net stride length for the locomotion in the pipe was 8.2 mm for each single cycle and the average speed in the pipe was 44 mm min^−1^.

Since more than half of the robot in length was soft segments and the battery integrated into the actuation module operated without a reduction in performance while the module underwent deformation, the robot could navigate through a curved pipe by bending its soft body. We tested the robot in a closed hexagon pipe consisting of six straight segments (*l*_straight_ = 315 mm) and six curved ones (*l*_straight_ = 330 mm, *R* = 300 mm) with 60° joint angles. After placing the robot into the pipe, we connected both ends of the hexagonal path to form a closed loop. The robot completed a single round trip with a travel distance of 3.87 m over 1 h and 20 min (Video S6, Supporting Information). The average speed for the whole round trip was 49.1 mm min^−1^ with a speed of 51.4 mm min^−1^ at the straight segments and 47.0 mm min^−1^ at the curved segments, respectively. The total energy that the fully assembled robot can store is 13.5 Wh and the average power consumption is 0.38 W, leading to a theoretical 35.6 h of operation time and 105.0 m of travel distance with a single charging of the system.

We tested the robot in a vertical pipe to climb up and down as the robot showed a strong grip on the pipe wall. Figure 5E shows the robot climbing up and down in the vertical pipe. The hydrostatic coupling of axial contraction and radial expansion allowed strong anchoring on the pipe wall, enabling climbing up against gravity while carrying all of its weight. The anchoring at the top with a single pod could hold the weight of the entire robot (≈950 g) while other modules were contracting. After all pods were contracted, all three pods except the last unspooled the motor tendon. The resilience of the pods enables them to push up on other pods, whilst the bottom one anchored against falling. By repeating the locomotion, the robot climbed up and down the vertical pipe with speeds of 25 and 50 mm min^−1^, respectively (Video S7, Supporting Information). We measured the force that the motor can generate within our power constraints to assess the robot's payload capacity. We found the maximum force that the motor generated was 42.5 N (Figure S9, Supporting Information), allowing the robot to lift an additional 1.4 kg on top of its own weight.

## 7 Discussion

Our work demonstrated that embodying a power source into motor driven‐tendon actuator modules provides an artificial muscle unit composable into robots capable of long duration, useful work. The adhesion between the ion membrane and polyurethane, we discovered, provided flexible hermetic sealing, solving a challenge in the fabrication of flexible redox flow batteries and enabling the production of the battery in various form factors and configurations. The anode pouch design, placing the anode inside a pouch made of ion membrane, allows for dual‐sided use, nearly doubling the power and capacity density compared to previous work (20.3 mW cm^−2^, 119 mAh cm^−2^, Table S1, Supporting Information). The anode pouch design also facilitated the use of multiple pouches in the pod, increasing the available total power and capacity within the robot. The robot fully functioned only with the embodied battery, supplying 0.78 W of power at voltages above 3.6 V, without the assistance of an additional battery. The battery, distributed throughout the entire body exhibited 13.5 Wh of total energy capacity, enabling prolonged operational duration.

The robot could crawl at speeds of 51.4 mm min^−1^ in a narrow pipe, even in the vertical pipe against gravity at speeds of 25 mm min^−1^. The speed of the robot was slower compared to that of robots powered through tethered connections (Table S2, Supporting Information). However, by eliminating the constraints by tethers, it was able to achieve a significantly extended operational range. The robot successfully navigated a closed hexagonal pipe loop with a single round trip of 3.8 m in 1 h and 20 min, consuming only 4% of the total energy capacity of its battery. This performance indicates that it can theoretically complete trips of over 100 m on a full charge, outperforming by at least more than ten times compared to existing untethered systems. The robot, which retained its compliant body even after integrating battery components, was able to deform to adapt to curved pipes and passed through with only about a 10% reduction in speed. Furthermore, as the modular robot consists of multiple pods, we can simply add an additional pod to extend the stride length per cycle. The addition of one pod to our current robot is expected to extend the stride length by 1.5 times, resulting in a 20% increase in speed after considering changes in cycle duration.

Although the robot in this work achieved a high energy capacity, there is still a challenge with the zinc‐iodide redox flow battery chemistry, as it is a hybrid flow battery with one side containing solid redox species. The electrode area on the anode for the zinc deposition during charging cycles may limit the capacity of the system even though there are still uncharged active species in the electrolytes. In our system, we adopted a pouch design that could be stacked within a single cathode chamber to accommodate more anode electrode area. Despite this effort, the capacity of a single module reached only 3387 mAh due to the limited anode area, even though the amount of electrolyte in the module could support a capacity of over 6000 mAh. Uneven electrochemical reactions between parallel‐connected anodes caused uneven zinc deposition and a drop in Coulombic efficiency, decreasing from 95% with a single pouch to 85% with three pouches connected in parallel, as verified through our experiments.

Our future work will focus on a fully liquid redox flow battery, where all active redox species are dissolved in the electrolyte. In such a system, the electrode area and capacity are completely decoupled, with the capacity determined solely by the volume of the electrolyte. This design normalizes uneven reactions at the electrode surface, as all active species return to the electrolyte, effectively addressing the challenges observed in hybrid systems. Notably, the polysulfide‐iodide battery, with its neutral pH, low cost, and high energy density, is emerging as a promising battery chemistry for soft robotics applications.^[^ ^47^ ^]^

## 8 Experimental Section

### The Pouch Anode Cell Fabrication

SIL30 (CARBON, INC.) is a 3D‐printable silicone polyurethane that has high stretchability and tear resistance. SIL30 also exhibits good chemical stability for electrolytes of zinc‐iodide flow batteries. The anode was composed of stainless‐steel mesh (Unique weaving wire, USA, 200 wires in.^−1^) and plain carbon cloth (AvCarb HCB 1071, 356 micron). The stainless‐steel mesh and the carbon cloth were cut into square shapes (28 × 28 mm^2^) using a laser cutter (Zing24, Epilog laser). The stainless‐steel mesh was placed between two layers of carbon cloth and secured together through sewing, as there were no rigid constraints in the battery cell for compression that would facilitate contact between the stainless steel and carbon cloth. The insulated copper wire was soldered onto the stainless steel and the solder was coated with epoxy resin to prevent undesired chemical reactions. SIL30 was 3D printed in a square frame shape with dimensions of 36 × 36 mm^2^, featuring a width of 3 mm and a thickness of 4 mm. The anode was placed within the interior space of the SIL 30 frame, and the terminal was created through the hole in the frame. The hole was thoroughly sealed with epoxy glue. The top and bottom surfaces of the frame used for the bonding area were cleaned with IPA. Ion exchange membranes (Nafion211, Dupont) were cut to the size of the SIL30 frame using a laser cutter. Once the ion exchange membrane was aligned with the frame, it was delicately compressed and rubbed to ensure a conformal contact.

### Creating a Full‐Cell Battery

As the pouch is a component comprised of the anode and anode enclosure that is made of the ion exchange membrane, the cathode composite was paired to create a full cell. Cathode composite was composed of tantalum mesh (Unique weaving wire, USA, 200 wires in.^−1^) and plain carbon cloth (AvCarb HCB 1071, 356 micron). We sewed together the tantalum mesh and the carbon cloth to bind and establish contact. The paired cathode had the same electrode area as the anode in the pouch. We chose the tantalum mesh as a current collector due to its chemical stability in the harsh oxidative environment experienced during the charging cycles. Unless otherwise indicated, 3 M of Zinc iodide (223883, Sigma‐Aldrich) solution including 10% of ethanol was used for both anolyte and catholyte. The anolyte was injected into the pouches, and the pouches and cathode were immersed in a chamber filled with the catholyte to form a full cell.

### Battery Performance Test

The assembled batteries using the battery testing system (Neware, BTS4000‐5V6A) were evaluated. The batteries were tested using galvanostatic mode with cutoff voltages of 1.45 and 0.4 V for charging and discharging cycles, respectively. To replenish the electrolyte during the test, the orbital shaker agitated the battery at 90 rpm of speed.

### Torque Measurement

The torque on the motor powered by the POD cells according to the number of PODs was measured. The metal geared motor (Pololu, 6V MP, 1000:1) that is used to build the robot was used for the torque measurement. The force gauge and the motor were mounted on the rack and connected through a Kevlar thread (50lb) and rubber band. The rubber band was added for the gradual increase of the tension. The POD cells were connected to the voltage boost for the step‐up and then connected to the motor. The diameter of the pulley on the motor for the torque measurement was 3 mm.

### Peel Strength Test for the Nafion Adhesion

T‐peel tests were conducted that measured the separation strength of two flexible strips to evaluate the bonding strength of the Nafion layer on polyurethane surfaces. The polyurethane substrates were rectangular strips, with a size of 50 mm x 15 mm x 1.5 mm. Nafion 115 (125 µm) was used to ensure that the Nafion layer can withstand the tensile stress during tests. The Nafion strips were cut into 40 mm x 10 mm pieces and then attached to the polyurethane strip, leaving 10 mm of unbonded ends. The unbonded ends were clamped in the test grip of the tensile machine (CellScale, Univert). Slow peeling speed is preferred to minimize energy dissipations on the stretchable substrate. The speed of the crosshead, where the gripper is installed, was chosen as 0.05 mm s^−1^.

### Fabrication of the Pod

The outer shell of the actuation module (pod) was 3D printed with SIL30 in a cylindrical shell structure with a curved wall on the side. The curved wall facilitates radial expansion of the side wall during axial contraction. A disk shape pouch cell was designed to fit in the pod and prepared them following the steps in Figure S6 (Supporting Information). The top and bottom surfaces of the pouch have a hexagon‐shaped mesh to provide mechanical support to the Nafion layer while allowing ion transport through it (with 75% open area). The pouches and bellow‐like structures were alternately stacked, making a watertight, flexible cavity in the center of the pod (Figure 4A). The anode terminals were made in the center cavity and the terminal wires were taken out through the cavity. The cathode was constructed using tantalum mesh (Unique weaving wire, 100 wires per inch, USA) and carbon cloth. Tantalum mesh was employed as a current collector due to its chemical inertness, preventing undesired side reactions such as metal dissolution. A single shared cathode (60 × 90 mm^2^) was placed around the pouches and then inserted into the cylindrical shell. The open face of the shell was closed with a pod cover and filled with the electrolyte by injection. The pod was inflated by injection for rapid radial expansion under contraction. The final diameter of the fully assembled pod was 60 mm. Two rigid end plates for the compression of the actuation module were 3D‐printed (3D systems, Problk‐10). One end plate with the motor holder and the other end plate were tied together using the Kevlar thread through the center cavity. One end of the thread, which is heading to the motor side, was fixed to the pulley (*R*: 5 mm), and then the pulley was installed on the shaft of the motor.

### Assembly of the Robot

Four identical pods with the method described above were prepared. Four pods were mechanically connected in series using the connecting parts that were printed on each end plate. For the control module, the module shell was 3D printed that has a semi‐cylinder shape. The printed circuit board, which includes a microcontroller (Arduino Pro Mini, 3.3V) and two dual‐channel motor drivers (DRV8833, Texas Instruments), was mounted onto this control module. The control module was then added to the end of the main body, to which four actuation pods were connected. The anode and cathode terminal wires in the four pods were electrically connected in series and powered the control module with 5.2 V. No other power supply source was added to the robot. The motor drivers and the motors on each module were wired through the internal cavity where the actuation thread was located. The block diagram of the system and the assembled robot are shown in Figure S8 (Supporting Information).

### Power Budget Optimization

The design and size of the robot were optimized by considering various factors, including the power required for the prime mover (motor) and control, the scalable size, and the minimum width necessary to maintain sealing. As shown in the Figure S9 (Supporting Information), the motor used can consume up to 0.75 W depending on the torque it generates. The microcontroller, Arduino Pro Mini 3.3 V 8Mhz, consumes 3.3 V × 5 mA = 0.015 W under normal active conditions. Two DRV8833 motor drivers consume 2 (3.3 V × 6 mA) = 0.04 W with no load. Additionally, the power dissipation in the H‐bridge, when the load current, *I*_load_, is 0.2 A, is calculated as *I*_load_^2^ × *R*_DS(on)_ = (0.2 A)^2^ × 0.45 Ω = 0.018 W, where *R*_DS(on)_ is H‐bridge on resistance. We anticipated a maximum total power consumption of approximately 0.8 W. It is confirmed that power density per cell area is maintained across different cell sizes (Figure S11, Supporting Information). The battery showed a power density of about 0.9 V × 150 mA/8 cm^2^ = 17 mW cm^−2^, requiring at least 47 cm^2^ of area to meet the power demands of the entire robot. Considering the losses during integration into the pod, four disk pouches with 8 cm^2^ of electrode area (ring shape, OD: 36 mm, ID: 16 mm) were placed into a single module, securing a total electrode area of 128 cm^2^. This configuration allowed to supply over 0.75 W of power at more than 3.6 V from the integrated batteries. Additionally, the excess electrode area contributes to an increase in the overall energy capacity of the robot as well.

## Conflict of Interest

The authors declare no conflict of interest.

## Supporting information



Supporting Information

Supplemental Video 1

Supplemental Video 2

Supplemental Video 3

Supplemental Video 4

Supplemental Video 5

Supplemental Video 6

Supplemental Video 7

## Data Availability

The data that support the findings of this study are available from the corresponding author upon reasonable request.
