# Supplementary material for: Soft, Modular Power for Composing Robots with Embodied Energy
Source: Adv Mater. 2025 Jan 2;37(7):2414872. doi: 10.1002/adma.202414872 (PMC11837883; doi:10.1002/adma.202414872)
Supplement: Supplementary file 1 — Supporting Information [file ADMA-37-2414872-s007.docx]

Supporting information for

**Soft, Modular Power for Composing Robots with Embodied Energy**

Chong-Chan Kim ^1^, Anunth Rao Ramaswami ^1^, Robert F. Shepherd ^1^*

^1^ Department of Mechanical and Aerospace Engineering

Cornell University

124 Hoy Road, Ithaca, NY 14850, USA

*Corresponding author, email: [rfs247@cornell.edu](mailto:rfs247@cornell.edu)

**This supporting information includes**

Figure S1 Design of the one-way valve on the frame and cyclic test of the battery cell

Figure S2 The hermetic sealing test of the bonding.

Figure S3 Bonding surface analysis through Raman spectroscopy

Figure S4 Bonding strength measurement using the T-peel test.

Figure S5. The humidity dependency of the bonding.

Figure S6 Fabrication of the pouch anode cells.

Figure S7 Fabrication of the pod battery.

Figure S8 Fully assembled robot.

Figure S9 The power consumption and the force of the motor.

Figure S10. The design of the setae for anisotropic friction.

Figure S11. The scalability test of the battery with various electrode areas.

Table S1. Comparison of the flexible power sources.

Table S2. Comparison of the worm-like soft robots.


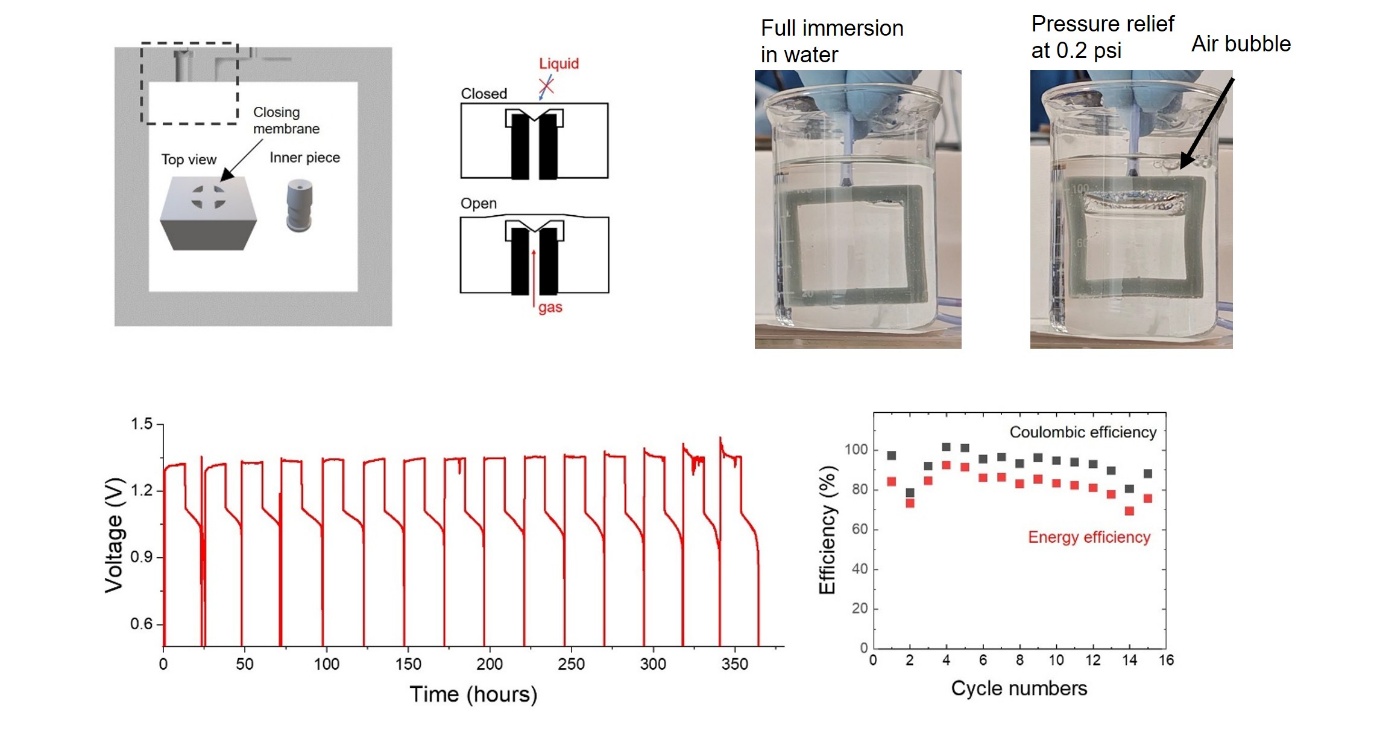


Figure S1. Design of the one-way valve on the frame and Cyclic test of the battery cell. Since the pouch anode cell is a sealed chamber, when hydrogen gas evolved during cycles and accumulated in the chamber, it pushed out the electrolyte through the Nafion, causing dehydration in the pouch and leading to poor cyclic capability. Previous work using a zinc iodide battery for a soft robotic system faced this dehydration issue after 10 cycles of 20 mAh cm^-2^ charging and discharging ^[28]^. We created a one-way valve on the anode pouch frame to release the hydrogen gas. The opening pressure was adjusted by the tension on the closing membrane. The valve released the gas when the internal pressure reached 0.2 psi, significantly improving the cyclic capability, achieving 15 cycles of 125 mAh cm^-2^ charging and discharging at 10 mA cm^-2^ over 360 hours.


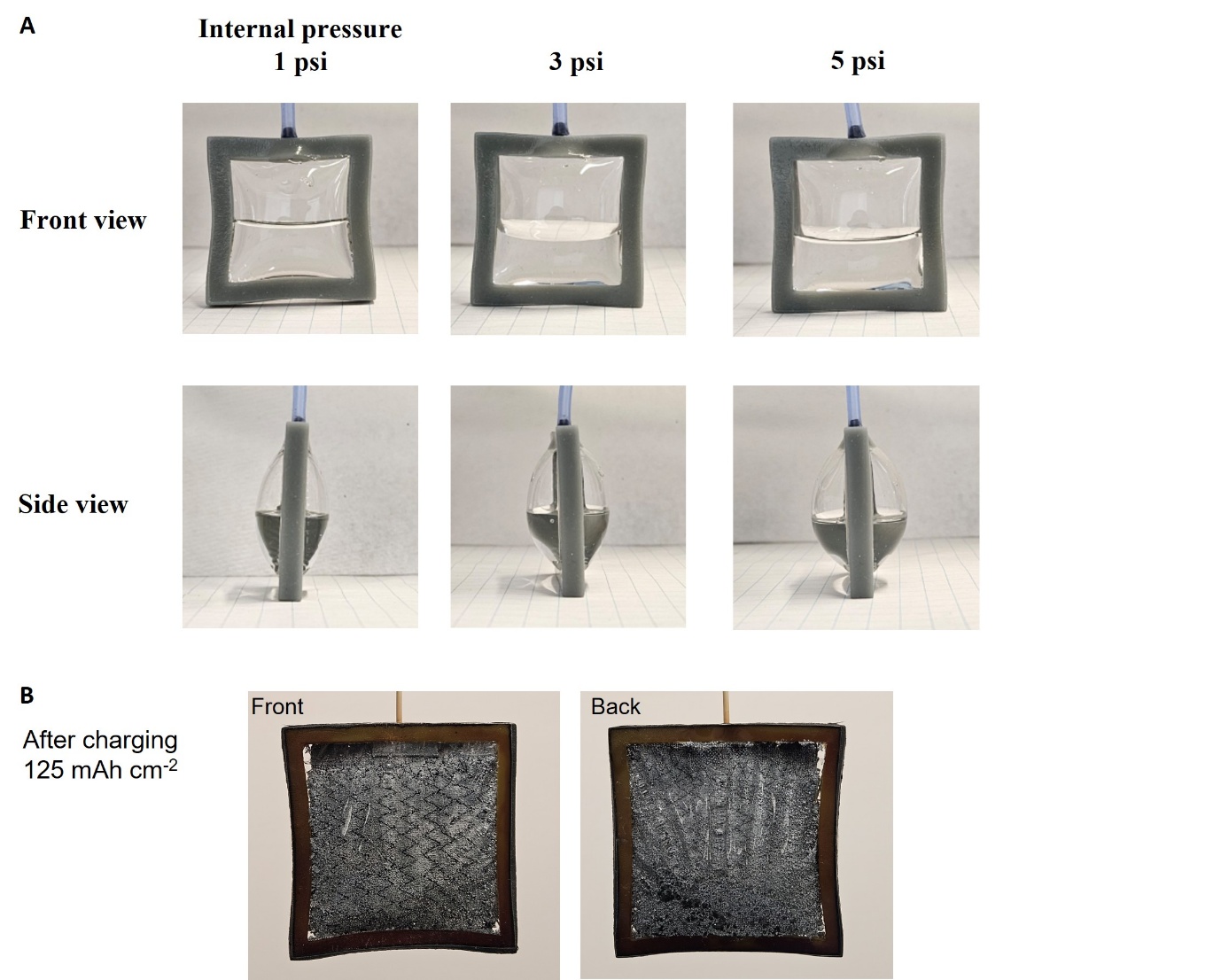


**Figure S2. The hermetic sealing test of the bonding.** We conducted a leakage test to verify the reliability of the bonding in providing a seal against internal pressure and ions. (A) We used square-shape polyurethane frame that we used in “Pouch anode cell”. The pouch was fully filled with water, and then air was introduced through a connected tube at a predetermined pressure. The test was conducted up to 5 psi, which is the point where severe permanent deformation occurs in the ion exchange membrane, but no delamination was observed. (B) The photograph of the anode pouch after charging of 125 mAh cm^-2^. The color of the anolyte remained clear, indicating it prevented cross mixing of the ions (No ion leakage)


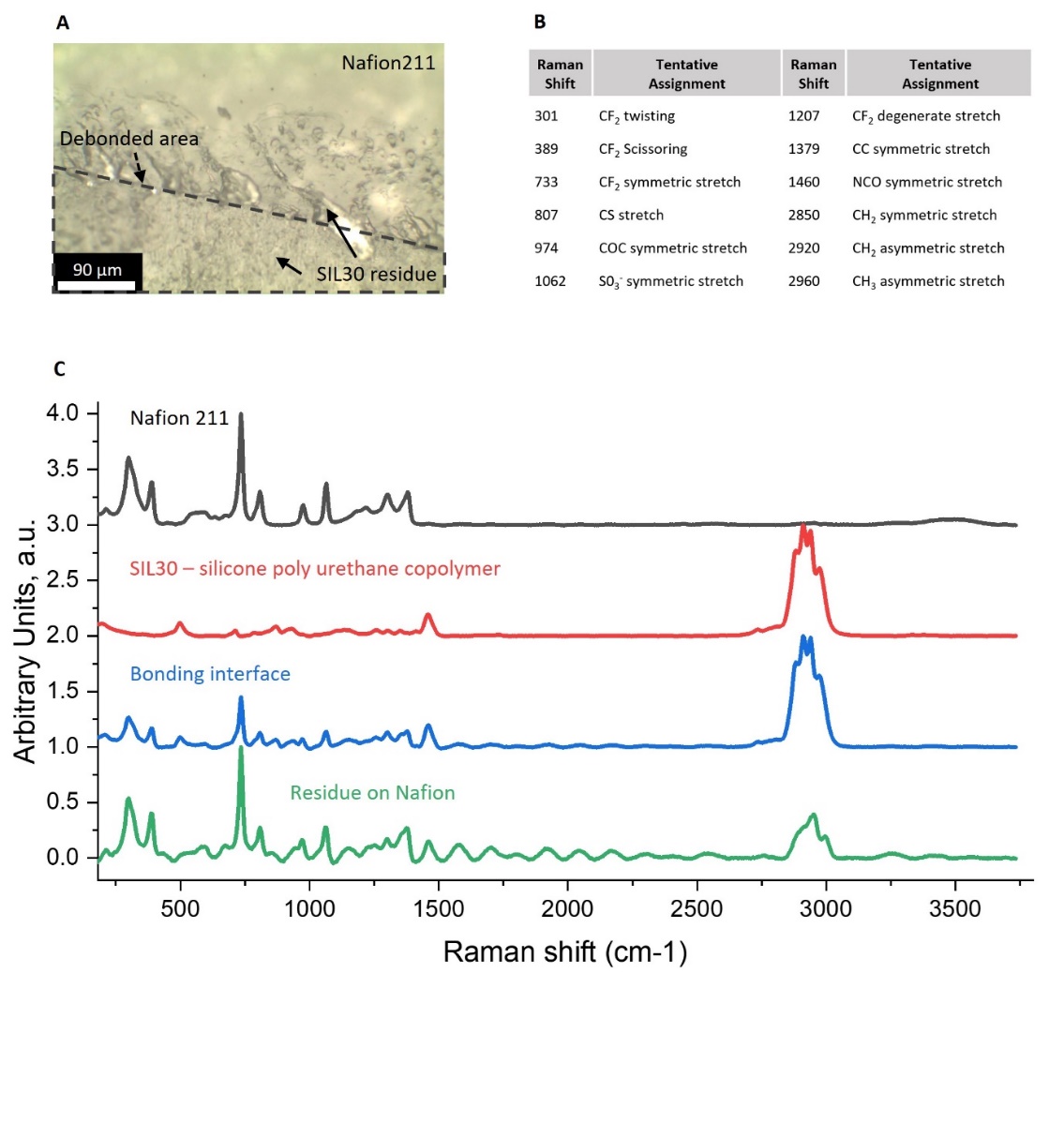


**Figure S3. Bonding surface analysis through Raman spectroscopy** Once the bonded surface was peeled off, the adhesion didn’t happen again on the Nafion membrane surface. To investigate the changes on the debonded surface, we analyzed it using Raman spectroscopy. Even in cases where no visible residue remained, we detected residue on the Nafion surface using microscope (A), and Raman spectroscopy confirmed that this residue was polyurethane. The residue exhibited mixed characteristic peaks of both Nafion and polyurethane (green curve in C). We also examined at the interface where bonding had originally formed, but no new bonding was observed, and characteristic peaks corresponding to both polyurethane and Nafion were observed


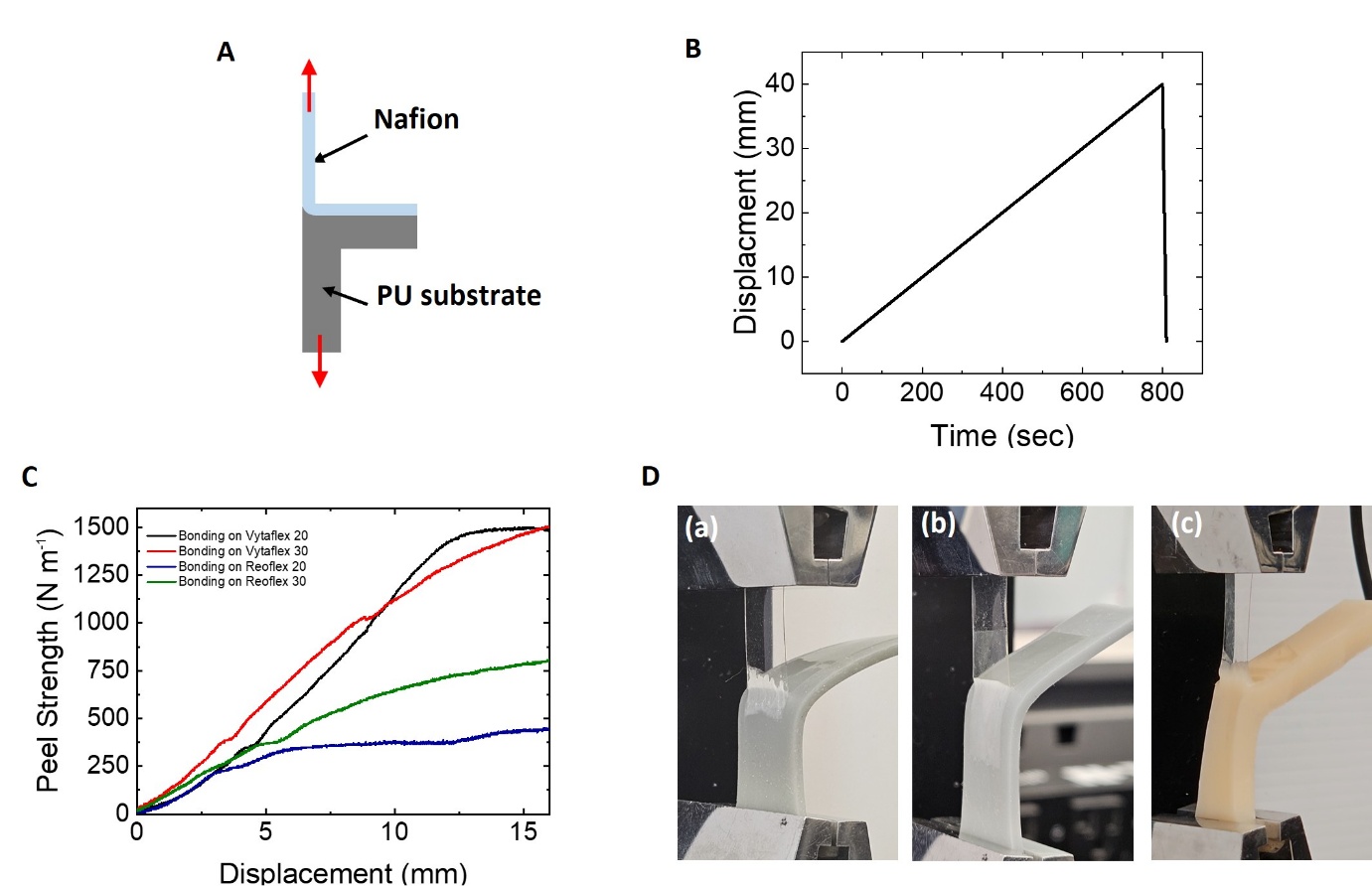


Figure S4. Bonding strength measurement using the T-peel test. (A) Diagram of the t-peel test. (B) Displacement curve of the crosshead of the tensile test. All tests were conducted with a constant peeling speed of 0.050 mm sec^−1^. (C) Peel strength of commercial polyurethane rubbers, (D) Photos of the T-peel test for (a) SIL30 (b) SIL30-wet (c) Vytaflex 30.


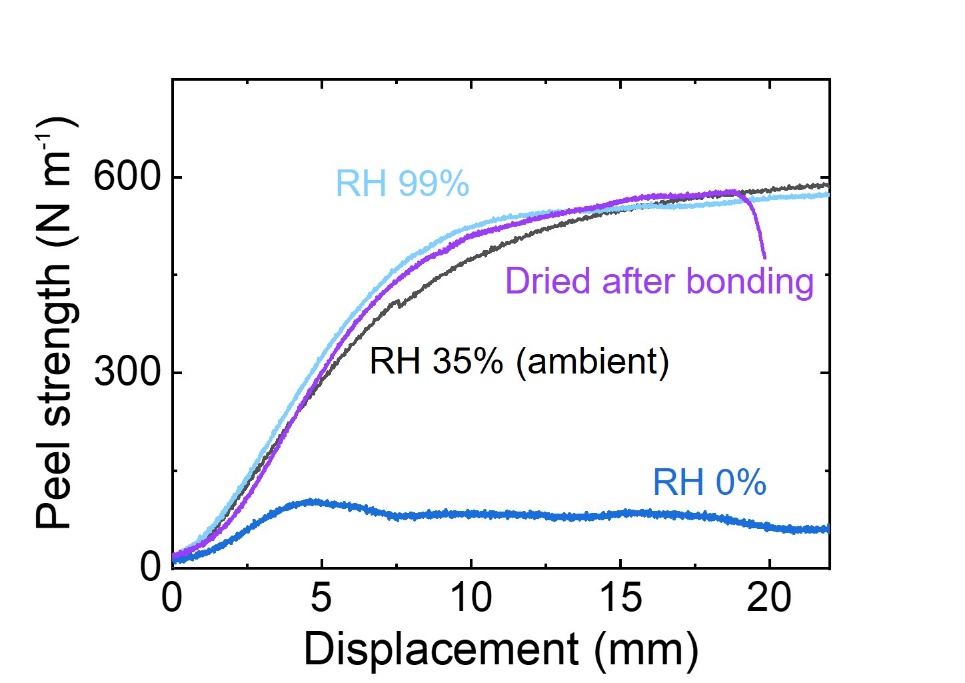


**Figure S5. The humidity dependency of the bonding.** The tests were conducted using specimens stored under three different humidity conditions. The ambient humidity of our laboratory space was 35%. Specimens representing the 0% condition were stored in a desiccator with a moisture-absorbing agent (DRIERITE desiccants, DRIERITE Co.) for 24 hours, reducing the moisture content to 0.005 mg L^-1^. This ensures a relative humidity of below 0.1%. Specimens for the 99% condition were stored in a container with a humidifier for 24 hours. The specimens used for the 99% humidity test were subsequently dried in a desiccator for one day before measuring the bonding strength again.


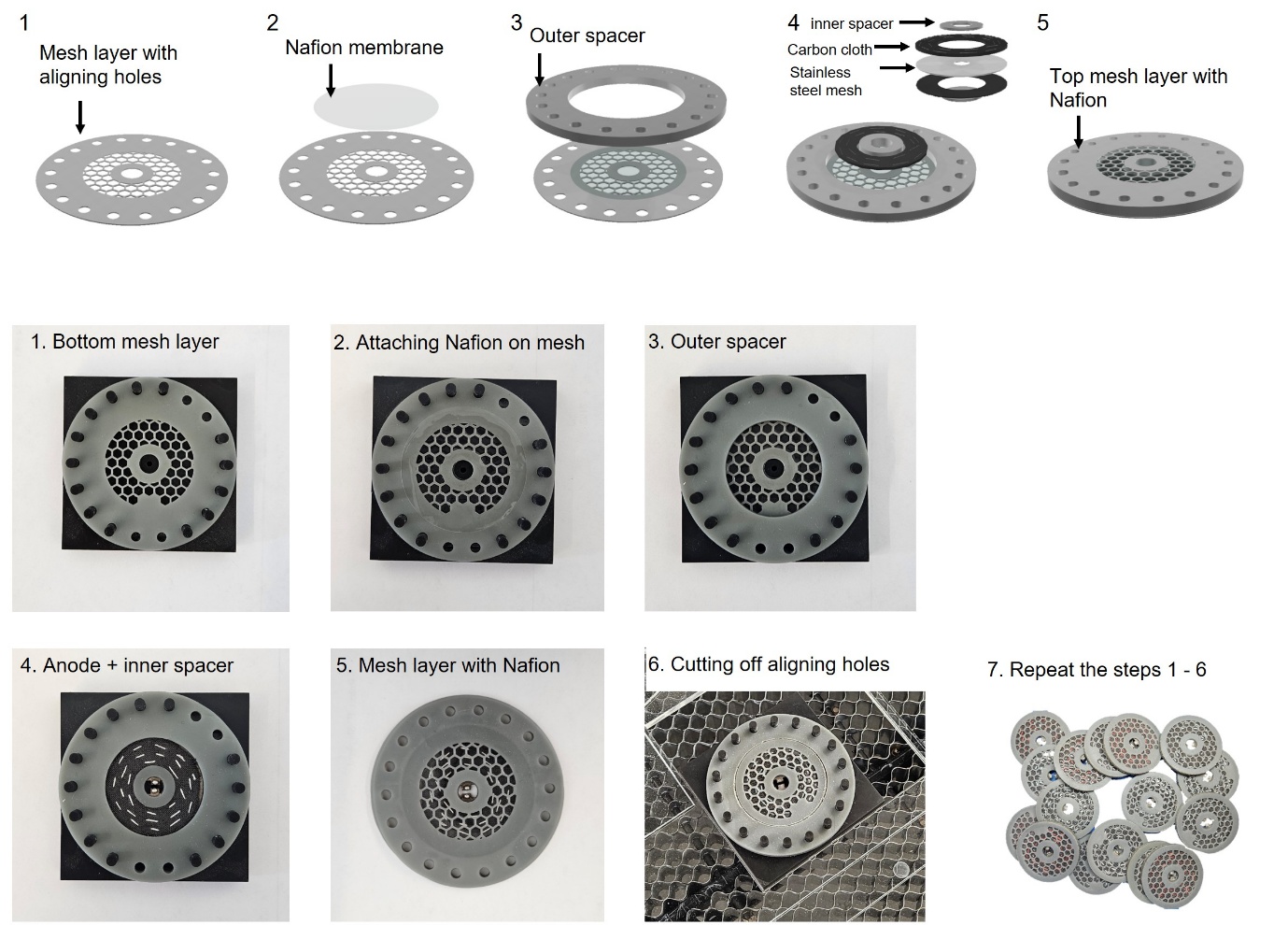


Figure S6. Fabrication of the pouch anode cells. To assemble the pouch anode cell, only the adhesion between Nafion and SIL30 was used. We 3D printed aligning holes together around the mesh for alignment of layers and preventing dimension change during the curing of SIL30. (1) We placed the mesh layer onto the alignment tool and (2) attached Nafion layer on the mesh. (3) We placed the outer spacer for anode electrode and (4) the pre-assembled anode composite (carbon cloth anode, stainless steel mesh current collector, inner spacer) on top of the mesh layer from step 2. The center hole was completely separated from the inner chamber that contains the anode and anolyte and used for wiring out the anode (5) We covered the inner chamber with the mesh layer to seal it. (6) We then cut off the aligning hole using the laser cutter.


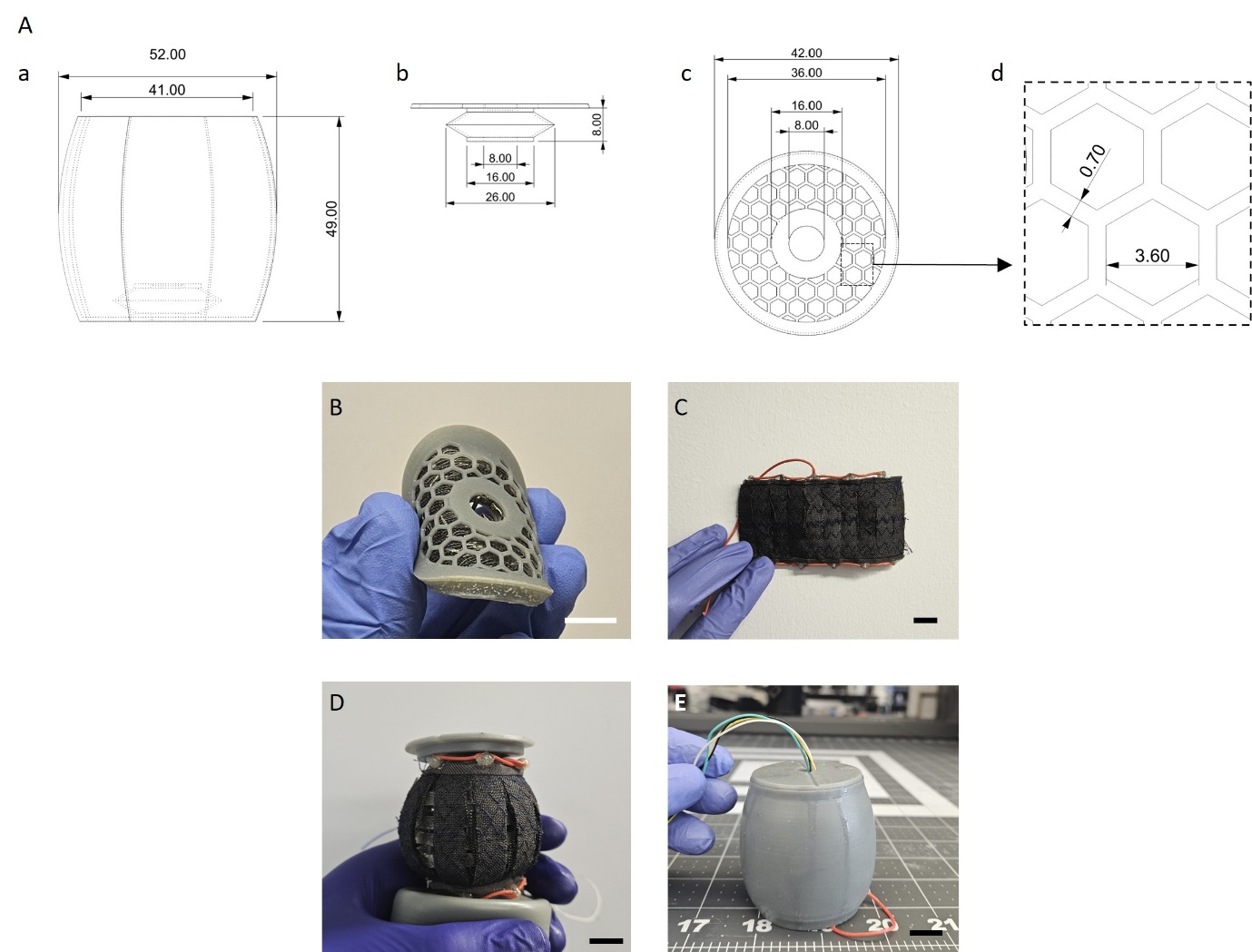


Figure S7. Fabrication of the pod battery. (A) Dimensions of the 3D printed parts, Outer shell of the pod, Cover of the pod, Pouch anode cell, Mesh in the pouch cell (B) Exploded view of the pod. (C) Flexible pouch anode cell. (D) Cathode in the pod. The total area of the cathode was 54 cm^2^ (6 cm x 9 cm). (E) The battery components in the pod. The cathode surrounds four pouch anode cells. (F) After assembling the inner components, we closed the outer shell of the pod with the top cover and injected the electrolytes.


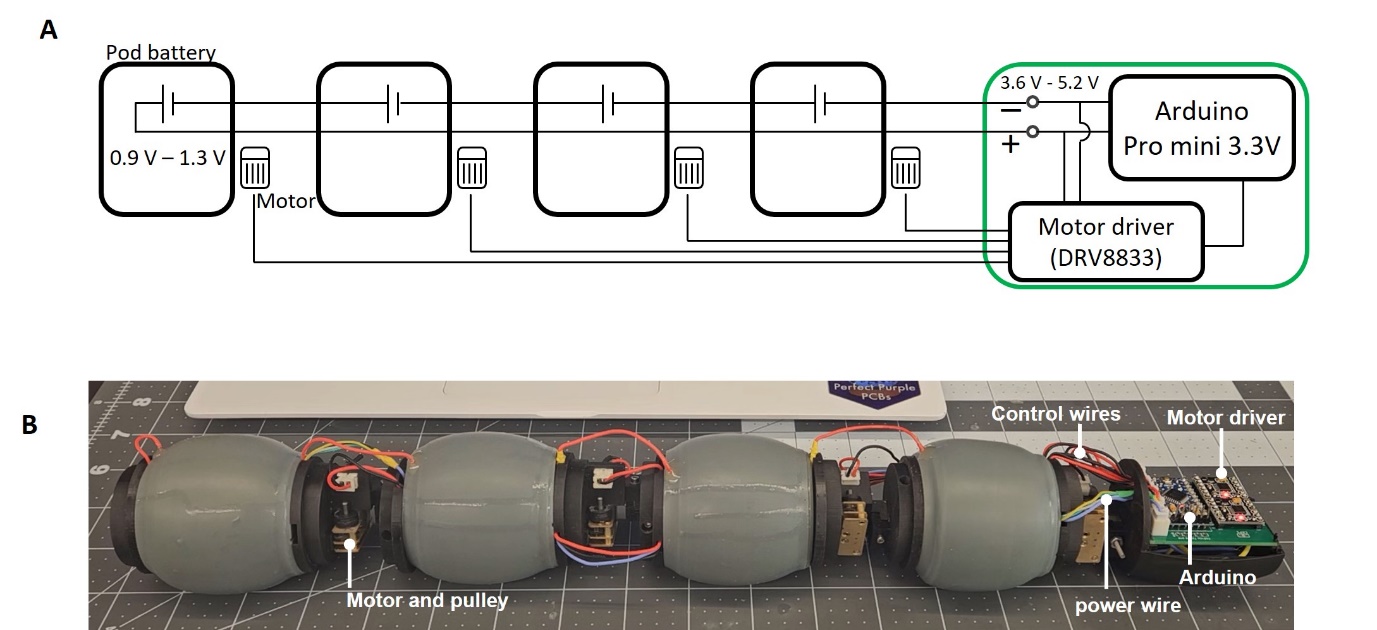


Figure S8. Fully assembled robot. (A) Block diagram of the system. Four series-connected batteries powered the microcontroller and the motor drivers. The motors on each actuation module were controlled by the microcontroller through the motor drivers. (B) The photo of the fully assembled robot.


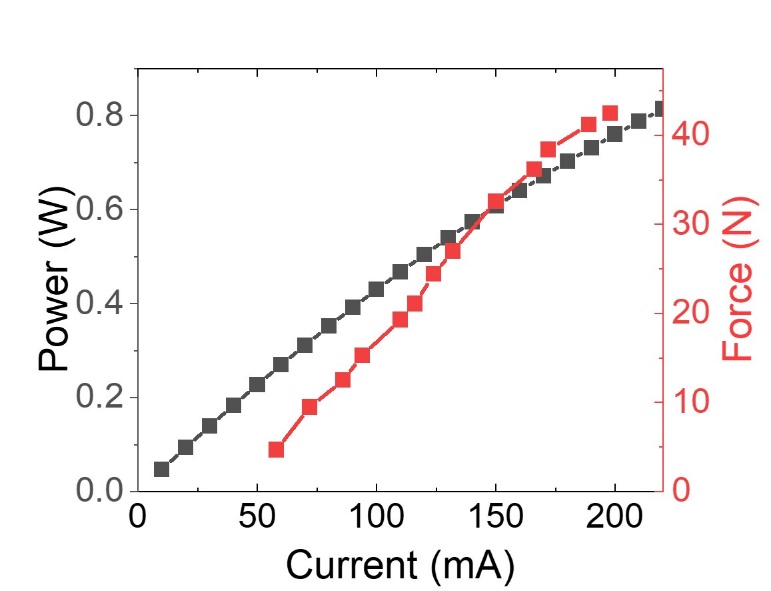


Figure S9. The power consumption and the force of the motor. The motor is rated for an instantaneous torque of 2.5 kgf∙cm, which corresponds to approximately 49 N when using a 5 mm diameter pulley. The maximum force within our target power was 42.5 N. The force required to deform the pod was 19.5 N, and the total weight of the worm robot was 950g. Therefore, the force required for the motor to climb vertically is 28.8N. We determine that the motor can support an additional payload corresponding to the remaining force of 13.7 N. Therefore, the motor can accommodate an extra payload of 1.4 kg.


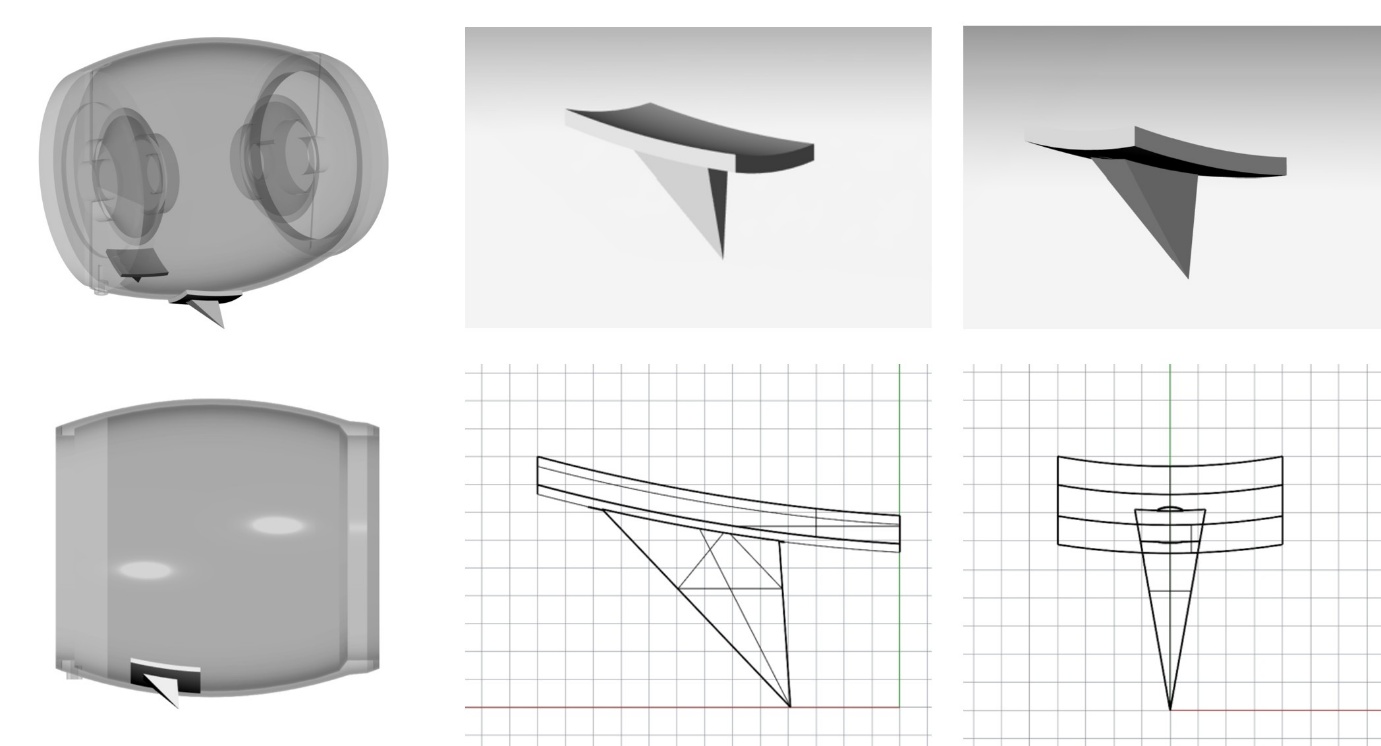


**Figure S10. The design of the setae for anisotropic friction.** Setae are composed of two components: a tetrahedral structure that generates anisotropic friction and a supporting base. The tetrahedral structure is designed to be solid and robust to support the weight of the pod. The base has a broad surface area to minimize deformation on the side wall of the module when the tetrahedron supports its weight. The setae are attached to the upper third of the module; this placement allows the stroke generated by contraction to be transferred more effectively to the ground. The grid size in the figure is 1 mm.


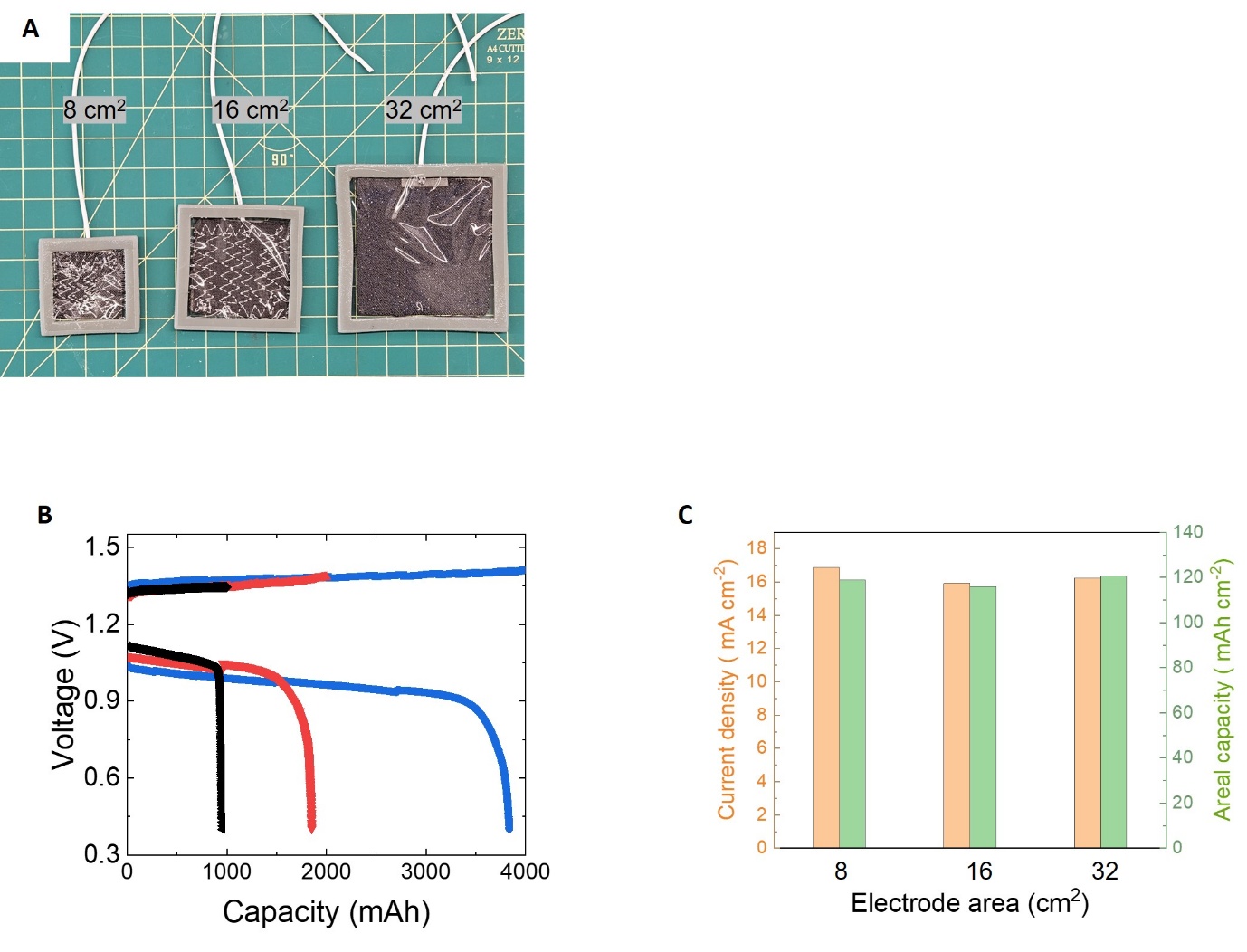


Figure S11. The scalability test of the battery with various electrode areas. (A) We investigated the scalability of the battery using three electrode areas (8 cm², 16 cm², and 32 cm²). Anode pouches were fabricated as described in The pouch anode cell fabrication and paired with two cathodes of the same size as each anode for the experiments. (B) Charging and discharging were performed at a current density of 10 mA cm⁻². All three cases achieved 92-95% Coulombic efficiency. (C) Despite the increase in electrode area, the current density and areal capacity remained consistent, confirming that larger electrodes can be used to increase capacity and power as needed.

| **Ref.** | **Type** | **Max. Pwr. density** | **Areal capacity** | **Size** |
| --- | --- | --- | --- | --- |
|  |  | **[ mW cm^-2^ ]** | **[ mWh cm^-2^ ]** | **[ cm^2^ ]** |
| This work | ZnI_2_ redox flow battery | 20.3 | 105.8 | 128 |
| 31 | Li-ion battery | 3.2 | 0.82 | 1×2 |
| 32 | Li-ion battery | 1.25 | 2.5 | 4×4 |
| 33 | Li-ion battery | 10 | 10 | 408 |
| 34 | Zinc-air battery | 4 | 100 | 1.5×4.5 |
| 35 | Zinc-air battery | 31.2 | 70 | 14×6.5 |
| 36 | Zinc-air battery | 60 | 12.76 | 4.5 |
| 37 | ZnI_2_ redox flow battery | 13.4 | 62 | 432 |
| 38 | Silver Zinc | 15 | 0.3 | 1×1 |
| 39 | Solar cell | 10 | - | 36 |
| 40 | Supercapacitor | 0.2 | 0.051 | 6.28 |
| 41 | Microbial fuel cell | 21.48 | 1.4 | 2×4 |
| 42 | Triboelectric harvester | 0.076 |  | 4×5 |

**Table S1. Comparison of the flexible power sources.** For cases where the current density and capacity were reported based on the mass loading of active materials, these values were converted to the power density per area and areal capacity using the mass loading per unit area as reported in the references.

**Table S2. Comparison of the worm-like soft robots.** H: horizontal pipe, V: vertical pipe

| **Ref.** | **Test environment** | **Actuation mechanism** | **Energy source** | **Duration, Capacity** | **Speed**  **[ mm s-1 ]** | **Weight**  **[ g ]** | **Length**  **[ mm ]** |
| --- | --- | --- | --- | --- | --- | --- | --- |
| This work | In-pipe locomotion (H, V) | Hydraulic  Tendon-driven | Untethered  (ZnI_2_ redox flow battery) | 35.5 h, 13.5 Wh | 0.86 (H), 0.42 (V) | 950 | 350 |
| 15 | In-pipe locomotion (H, V) | Pneumatic | Tethered | - | 6 (V) | 96 | 64 |
| 16 | In-pipe locomotion (H, V) | Pneumatic | Tethered | - | 33 (H), 30 (V) | 57 | 153 |
| 17 | In-pipe locomotion (H) | Linear actuator, motor | Tethered | - | 8 mm cycle^-1^ | 231 | 150 |
| 18 | In-pipe locomotion (H, V) | Pneumatic | Tethered | - | 5.05 (H), 4.23 (V) | 85.4 | 50 |
| 19 | In-pipe locomotion (H, V) | Pneumatic | Tethered | - | 4 (H), 1.67 (V) |  | 130 |
| 13 | Crawling on a flat surface | Pneumatic | Untethered  (Li-ion battery) | 0.5 h, 0.55 Wh | 0.28 | 200 | 210 |
| 22 | Crawling on a flat surface | Magnetic induction heating | Untethered  (external magnetic field) | - | 0.15 | 0.6 | 40 |
| 12 | Crawling on a flat surface | Shape-memory alloys | Untethered  (Li-ion battery) | 0.17 h ,0.63 Wh | 0.495 | - | 200 |
| 14 | Crawling on a flat surface | Shape-memory alloys | Untethered  (Li-ion battery) | 1.2 h, 1 Wh | 0.3 | 4.2 | 150 |
| 20 | In-pipe locomotion (H, V) | Pneumatic | Untethered,  (Li-ion battery) | 2 h | 1.27 (H), 0.39 (V) | 3,050 | 1,200 |
| 21 | In-pipe locomotion (H) | Linear actuator, motor | Untethered,  (Li-ion battery) | 36 Wh | - | 1,300 | 110 |
